# Menthol tobacco use is correlated with mental health symptoms in a national sample of young adults: implications for future health risks and policy recommendations

**DOI:** 10.1186/s12971-015-0066-3

**Published:** 2016-01-08

**Authors:** Amy M. Cohn, Amanda L. Johnson, Elizabeth Hair, Jessica M. Rath, Andrea C. Villanti

**Affiliations:** The Schroeder Institute for Tobacco Research and Policy Studies at Truth Initiative, 900 G Street, NW, Fourth Floor, Washington, DC 20001 USA; Department of Oncology, Georgetown University Medical Center, Cancer Prevention and Control Program, Georgetown Lombardi Comprehensive Cancer Center, 3800 Reservoir Road, NW, Washington, DC 20057 USA; Department of Evaluation Science and Research, Truth Initiative, 900 G Street, NW, Fourth Floor, Washington, DC 20001 USA; Department of Health, Behavior and Society, Johns Hopkins Bloomberg School of Public Health, 615 North Wolfe Street, Baltimore, MD 21205 USA

**Keywords:** Menthol, Mental health, Young adults, Depression, Anxiety

## Abstract

**Background:**

Depression and anxiety are correlated with greater nicotine dependence, smoking persistence, and relapse back to smoking after a quit attempt. Menthol cigarette smoking, which is prevalent in young adults, is associated with nicotine dependence, progression to regular smoking, and worse cessation outcomes than non-menthol smoking. Few have established a link between menthol tobacco use, beyond just smoking, with mental health in this high-risk age group. This study examined the association of menthol tobacco use to anxiety and depression in a national sample of young adults.

**Methods:**

Data were from Waves 1 through 7 (n = 9720, weighted) of the Truth Initiative Young Adult Cohort, a national sample of men and women aged 18 to 34 assessed every 6-months. Demographics, past 30-day use of non-menthol and menthol tobacco products, and current alcohol, marijuana, and other drug use were assessed among those with depression and anxiety.

**Results:**

Thirty nine percent of current tobacco users used menthol as their preferred brand. Using a cross-sectional analysis (collapsed across waves), past 30-day menthol tobacco was uniquely associated with greater odds of both depression and anxiety, beyond the effects of demographic and substance correlates and non-menthol tobacco product use.

**Conclusions:**

Menthol is disproportionately used among young adults tobacco users with mental health problems, above and beyond the impact of a variety of other mental health and tobacco use risk factors. Findings suggest a strong link between menthol tobacco use and poor health outcomes. Policies should be developed to deter menthol tobacco use in vulnerable groups.

## Background

Menthol comprises nearly a third of the tobacco market. It’s minty taste and cooling and anesthetizing properties have been posited to reduce the harshness of smoke inhalation, may contribute to tobacco use and smoking initiation in young people [1–4], and may prevent tobacco users from quitting [5–7]. While recent data show decreases in the prevalence of non-menthol cigarette smoking [6, 8] there has been an increase in menthol cigarette use in young adults during this same time period [9]. Menthol has been proposed as an important factor in the transition from initiation to regular tobacco use during young adulthood [3, 4].

Menthol may increase the addictive potential of tobacco use via multiple mechanisms, including biological, genetic, environmental, and dispositional [10–16]. Menthol tobacco use may be maintained through its association with positive, pleasurable, and other hedonistic responses to nicotine. This may attract new users [3, 4] and facilitate greater ingestion of harmful tobacco constituents [11]. Further, the highly salient positive sensory properties of menthol may enhance the overall rewarding effects of nicotine reinforcement [12, 17], thereby strengthening the uptake of cigarette or other nicotine-containing tobacco products among emergent users. This then enhances the addiction potential of menthol tobacco products, and may hinder success at quitting later on [18].

Psychiatric distress, including depression and anxiety, is linked to tobacco use, greater nicotine dependence, smoking persistence, and poor smoking cessation outcomes [19–22]. Similarly, menthol cigarette smoking has consistently been associated with more severe nicotine dependence [23] and lower quit rate success than non-menthol smokers [24–26]. A paucity of published research, however, has focused on the link between mental health factors and menthol cigarette and other tobacco product use in young adult populations, despite the exceedingly high rates of menthol tobacco use in this age group. Of the few studies published linking menthol use to mental health, findings show that the prevalence of menthol cigarette smoking is high among those with severe psychological distress compared to those without [11, 27, 28] and that menthol smokers report worse general mental health than non-menthol smokers [29, 30]. One recent population-based study of young adults found that menthol cigarette smoking was correlated with marijuana use and binge drinking [31], two health-risk behaviors that are also robustly linked to psychiatric distress [32–37]. One explanation for the association between menthol cigarette smoking and mental health problems could be due to the cooling sensation of menthol, which has a mildly arousing and stimulating effect [38], and may dampen frequent or chronic dysphoric mood often present with certain mental illnesses. Indeed, prior research shows that menthol cigarettes are used to enhance drug-related high and to sustain euphoric mood states [39]. Other research suggests that the nicotine in tobacco, more generally, is used to self-medicate mood disorders [36, 40]. If this was the case, then menthol tobacco use would not be differentially associated with mental health problems, compared to non-menthol tobacco use. Taken together, the stimulating and arousing properties of menthol in nicotine-containing tobacco products might be attractive to young adults with mental illnesses, perhaps to self-medicate and cope with negative mood states. If menthol increases the subjective effects of nicotine on mood improvement, one might expect greater uptake of menthol tobacco products in young adults with mood problems than those without.

In sum, mental health problems and menthol cigarette smoking have been robustly linked to smoking persistence, nicotine dependence, and poor smoking cessation rates [20, 41]. Further, a high prevalence of menthol cigarette use has been documented among young adult tobacco users [8, 42, 43]. As such, it would be important to determine whether menthol tobacco use is associated with increased mental health problems in this age group. Such findings may explain, in part, why menthol cigarette use is correlated with smoking initiation and progression in young people – it is being used to self-medicate or cope with stress and emotional problems. Further, because young adults use a variety of tobacco products, not just cigarettes [44], it would be important to examine the association of all types of menthol tobacco products, beyond cigarettes, to mental health problems. This study used a national sample of over 9600 young adults to examine the prevalence of depression and anxiety as a function of menthol tobacco product use, after adjusting for demographic and psychosocial risk factors known to impact both mental health and tobacco use (i.e., alcohol, drug use). We hypothesized that menthol tobacco use would be associated with increased mental health risk, above and beyond the impact of other robust mental health and tobacco use risk factors. Additionally, because this study examines the association of menthol tobacco use with depression and anxiety, above and beyond other mental health and tobacco use risk factors, we also describe the links between these risk factors and the outcomes of interest, to situate our primary hypothesis in context. Such findings would suggest that removal of menthol tobacco products from the tobacco marketplace may prevent initiation and progression to regular use among particularly vulnerable groups of incipient users.

## Methods

### Sample

Data were from Waves 1 through 7 of the Truth Initiative Young Adult Cohort, a national sample of men and women aged 18 to 34 drawn from the GfK KnowledgePanel®. Waves were assessed at approximately 6-month intervals, from June 2011 (Wave 1) to October 2014 (Wave 7). KnowledgePanel® is an online panel of adults ages 18 and older that covers both the online and offline populations in the U.S (http://www.gfk.com/products-a-z/knowledgepanelr-northamerica/). The panel was recruited via address-based sampling, a probability-based random sampling method that provides statistically valid representation of the U.S. population, including cell phone-only households. Households without internet access were provided a free netbook computer and internet service to reduce response bias in typical online survey samples. African-American and Hispanic individuals were oversampled. The validity of this methodology has been reported previously [45–50].

The panel recruitment rate ranged from 14-15 % across all waves [51]. In 64-66 % of these identified households, one member completed a core profile survey in which the key demographic information was collected (profile rate). For this particular study, only one member per household was selected at random. The response rate ranged from 29-55 % and the cumulative response rate across all surveys ranged from 4-7 %, which is consistent with other published studies [52, 53].

After Wave 1, the sample for each wave consisted of people who completed the previous wave plus a refreshed sample to replace individuals lost to follow-up and to ensure the sample did not age out of the cohort. This study was approved by the Independent Investigational Review Board, Inc. for Waves 1-3 and Chesapeake Institutional Review Board, Inc. for Waves 4-7. Additional details on survey methodology have been published elsewhere [54]. The present analysis focused on a subset of 9658 (weighted *n* = 9720) young adults aged 18 to 34 at study entry who answered questions on mental health and contributed 26,378 (weighted *n* = 26,587) cases/observations over seven waves of data collection.

### Measures

#### Mental health symptoms

As the primary outcomes, two variables were created to assess symptoms consistent with current (past 2 weeks) depression and anxiety from the two-item Patient Health Questionnaire (PHQ-2) [55, 56] or the two-item Generalized Anxiety Disorder (GAD) scale [57, 58], respectively. Using a 4-point scale (0 = “not at all” and 3 = “nearly every day”), PHQ-2 queries about the frequency of depressed mood and loss of interest in pleasant activities, while the two-item GAD queries about the frequency of uncontrollable worry and feelings of anxiety. According to validated scoring guidelines [56, 58], individuals who received a score at or above the cut-off (≥3) were coded as having either depression or anxiety at that particular wave.

#### Tobacco use

At each wave, participants were asked about past 30-day use of eleven tobacco products including: cigarettes, cigars, pipe, little cigars/cigarillos (LCCs), e-cigarettes, chewing tobacco, dip/snuff, snus, dissolvable products, hookah/shisha, and other nicotine products (i.e., gum, patches, lozenges). Participants who reported use of any tobacco product in the past month were defined as past 30-day tobacco users in that particular wave. For each product used, participants were asked to identify the typical brand used in the past 30 days and whether it was menthol-flavored. Participants who reported that their typical brand was mentholated were defined as a current menthol tobacco user in that particular wave. Following established methods [59, 60], examples of usual brands were included in the question to further assist with the query. Past 30-day non-menthol tobacco use and past 30-day menthol tobacco use are wave-specific variables. Thus, an individual could be both a past 30-day tobacco user and not a past 30-day menthol tobacco user, depending on the wave.

#### Alcohol, marijuana, other drug use

Current alcohol use was assessed differently for Waves 1-6 versus Wave 7. In waves 1-6, all participants were asked about the frequency of their current use, with response options “every day,” “some days,” and “not at all.” Those who reported using alcohol “some days” or “every day” were defined as current users in that particular wave. For wave 7, current alcohol use was determined by two items. The first item asked about frequency of drinking alcohol in the past year (“never,” “monthly or less,” “2-4 times per month,” “2-3 times per week,” “4 or more times per week”). Those who reported any use of alcohol were then queried about the frequency of use in the past 30 days, with respondents using ≥1 days in the past month defined as current users. Those with missing data (<2 %) were defined as non-current users in that particular wave.

For current marijuana and other drug use (cocaine, ecstasy, meth, etc.), participants were asked about the frequency of their use for each substance, with response options “every day,” “some days,” and “not at all,” similarly across all waves. Those who reported current use some days or every day were defined as current users in that particular wave. Those with missing data or who reported “not at all” (<2 %) were defined as non-current users in that particular wave.

#### Demographics

Participants provided information on age at study entry (grouped as 18-24 and 25-34), gender, race/ethnicity (White, non-Hispanic; Black, non-Hispanic; Other, non-Hispanic; and Hispanic), highest education completed (less than high school, high school, and some college or greater), and financial situation (does not meet basic needs, just meets basic needs, meets needs with a little left, and lives comfortably). Financial situation was assessed at Waves 1, 3, 5, and 7; and was imputed using a carry-forward approach from the previous wave for waves 2 and 4 and a carry-backward approach for wave 6 [61, 62].

### Analyses

Analyses were performed using Stata/SE 13.1 (StataCorp, 2014) in April 2015. Post-stratification weights were used to offset non-response or non-coverage bias and to produce nationally representative estimates specific to each wave. Our main hypothesis was not focused on the impact of menthol use on changes in depression and anxiety over time. Preliminary analyses of the intra-class correlations (ICCs) did not show significant variations over time in prevalence of depression or anxiety, therefore data were collapsed across the 7 study waves. That is, because prevalence estimates of depression and anxiety at each time point were not statistically different, we collapsed data across all observations at each wave. To account for clustering of individual responses over time, participant ID was set as the primary sampling unit in all analyses and study wave was included as a covariate in analyses. The robust Poisson regression method was used to adjust for inflation of the odds ratio, given that the prevalence of the outcomes were higher than 10 % (i.e., the recommended standard) of the total sample [63].

We calculated the prevalence of anxiety and depression in the sample. Bivariate modified Poisson regressions were then conducted to examine correlates of anxiety and depression. Next, we examined overall changes in prevalence ratios of the association of past 30-day menthol tobacco use on anxiety or depression after covariate adjustment. To do this, three separate multivariable modified Poisson regression models were conducted (separately for each outcome) that included demographics in step 1; demographics, tobacco, and substance use variables entered in step 2; and the addition of menthol-flavored tobacco use in step 3.

Because data are collapsed across waves, modified Poisson regression prevalence ratios represent the association of a person at a specific time point, reporting that they were a menthol or non-menthol user (for example). All models controlled for study wave and missing data were handled with modelwise deletion per Stata’s survey procedures. Missing data values are reported in Table 1.

**Table 1:** Weighted sample characteristics by mental health status, across all waves (1-7) in n = 9720 young adults using crude unadjusted Poisson regression

		Total	Anxiety	Depression
		Weighted N = 26,587	Weighted N = 3,870	Weighted N = 3,549
	Overall		14.70 %	13.43 %
Age	18-24 (referent)	60.26 %	14.49 %	13.04 %
	25-34	39.74 %	15.02 %	14.02 %
Sex	Male (referent)	49.68 %	12.36 %	12.19 %
	Female	50.32 %	17.01 %**	14.65 %*
Race	White, non-Hispanic (referent)	58.26 %	14.37 %	11.80 %
	Black, non-Hispanic	13.14 %	15.87 %	16.62 %**
	Other, non-Hispanic	8.35 %	16.42 %	15.91 %*
	Hispanic	20.25 %	14.19 %	15.05 %*
Education	Less than high school	11.81 %	18.37 %*	16.64 %**
	High school	27.13 %	15.33 %	15.69 %**
	Some college or greater (referent)	61.05 %	13.72 %	11.81 %
Financial Situation	Don’t meet basic expenses	7.85 %	26.90 %**	26.31 %**
	Just meet basic expenses	30.97 %	18.27 %**	16.42 %**
	Meet needs with a little left (referent)	37.84 %	11.86 %	10.64 %
	Live comfortably	23.34 %	9.85 %*	9.16 %
Past 30-day tobacco use	No (referent)	75.57 %	12.59 %	11.63 %
	Yes	24.43 %	21.23 %**	19.00 %**
Past 30-day menthol tobacco use	No (referent)	90.23 %	13.60 %	12.40 %
	Yes	9.77 %	24.96 %**	22.93 %**
Current alcohol use	No (referent)	42.55 %	13.92 %	13.36 %
	Yes	57.45 %	15.28 %**	13.48 %
Current marijuana use	No (referent)	87.43 %	13.19 %	12.06 %
	Yes	12.57 %	25.27 %**	22.99 %**
Current other drug use	No (referent)	97.54 %	13.96 %	12.77 %
	Yes	2.46 %	44.14 %**	39.65 %**

Note. Unweighted respondent n = 9658. Data represent row percents. Missingness for financial situation due to lagged wave assessment was 7.0 %. Both 30-day tobacco use and 30-day menthol tobacco use were assessed out of the full study sample. Those with missing data on alcohol, tobacco, marijuana, and other drug use (<2 %) were defined as non-current users

*p < 0.05 **p <0.001

## Results

### Prevalence and correlates of anxiety and depression

The sample (see Table 1) was composed of equal numbers of men and women, with the majority aged 18- 24 (60 %), White, non-Hispanic (58 %), having at least some college education (61 %), and a little over a third met financial needs with a little left over (38 %). Overall, 13 % of the sample reported symptoms consistent with current depression and 15 % reported symptoms consistent with current anxiety. Almost half (45 %) of those who reported depression or anxiety endorsed symptoms consistent with both mental health issues. Of the total sample, 58 % were current alcohol users, 24 % were past 30-day tobacco users, 13 % were current marijuana users and 2 % were current users of other drugs. Of those who were past 30-day tobacco users, 39 % used menthol as their preferred brand (of at least one tobacco product).

A sub-analysis (not reported in Table 1) was conducted to examine menthol use across race. Among past 30-day tobacco users in the study sample, 67 % of Black respondents reported current use of menthol tobacco, followed-by 45 % of non-Black, non-Hispanic, 44 % of Hispanic, and 32 % of White respondents (*p <* 0.001).

Crude prevalence ratios from bivariate modified Poisson regression models showed that females, those with less than a high school education (vs. some college or greater), and those with financial situations that at most just met basic expenses (vs. meeting needs with a little left) were more likely to report depression or anxiety (all *p’*s < 0.05).

Race, education, and financial situation varied across the mental health outcomes. Non-White respondents were more likely to report depression, but not anxiety (all *p*’s <0.05). Those with a high school education (vs. some college or greater) were significantly more likely to report symptoms of depression, but not anxiety (*p* <0.001). Those who reported living comfortably (vs. meeting needs with a little left) were less likely to report anxiety (*p* =0.020). Depression appeared unrelated to financial situation (*p* =0.071). Age was not associated with depression or anxiety (*p*’s 0.30 - 0.60).

Current users of tobacco, marijuana, and other drugs were more likely to report depression and anxiety compared with non-users (all *p*’s <0.001). Current alcohol users were more likely to report symptoms of anxiety (*p* =0.014), but not depression (*p* =0.404). Almost 25 % of respondents who reported past 30-day menthol tobacco use also reported anxiety or depression (Table 1). Within the full sample, however, only ~10 % of respondents reported past 30-day menthol tobacco use.

### Stepwise modified Poisson regression models

Table 2 shows correlates of current anxiety across all step-wise three models. Adjusting for all covariates in the final model (Model 3), past 30-day use of any menthol tobacco product emerged as a significant correlate of current anxiety. Female gender and lower financial means remained significantly associated with increased odds of anxiety after including all variables in the model (Model 3). Past 30-day tobacco use, current marijuana use, and current other drug use also remained significantly correlated with anxiety in the final model.

**Table 2:** Stepwise modified Poisson regression models of menthol use associated with current anxiety among n = 9720 (weighted) young adults

	Model 1			Model 2			Model 3		
	Demographics			Demographics, tobacco, and substance use			Demographics, tobacco, substance use and menthol use		
Predictors	APR	(95 % CI)	*p*	APR	(95 % CI)	*p*	APR	(95 % CI)	*p*
Age									
18-24	Reference			Reference			Reference		
25-34	1.03	(0.91, 1.15)	0.679	1.03	(0.92, 1.16)	0.609	1.03	(0.92, 1.16)	0.598
Gender									
Male	Reference			Reference			Reference		
Female	1.32	(1.18, 1.49)	0.000	1.41	(1.25, 1.58)	0.000	1.40	(1.24, 1.57)	0.000
Race									
White, non-Hispanic	Reference			Reference			Reference		
Black, non-Hispanic	0.95	(0.77, 1.16)	0.603	0.95	(0.78, 1.17)	0.644	0.94	(0.76, 1.14)	0.515
Other, non-Hispanic	1.17	(0.97, 1.42)	0.106	1.19	(0.99, 1.43)	0.071	1.18	(0.98, 1.42)	0.087
Hispanic	0.92	(0.80, 1.07)	0.284	0.96	(0.83, 1.11)	0.594	0.96	(0.83, 1.10)	0.532
Education									
Less than high school	1.17	(0.97, 1.42)	0.106	1.07	(0.90, 1.29)	0.439	1.07	(0.89, 1.28)	0.469
High school	1.02	(0.89, 1.16)	0.799	0.98	(0.86, 1.12)	0.748	0.98	(0.86, 1.12)	0.754
At least some college	Reference			Reference			Reference		
Financial Situation									
Does not meet basic expenses	2.19	(1.85, 2.61)	0.000	2.01	(1.69, 2.38)	0.000	2.00	(1.69, 2.37)	0.000
Just meets basic expenses	1.53	(1.35, 1.73)	0.000	1.48	(1.32, 1.68)	0.000	1.49	(1.32, 1.68)	0.000
Meets needs with a little left	Reference			Reference			Reference		
Lives comfortably	0.83	(0.71, 0.97)	0.022	0.84	(0.72, 0.97)	0.021	0.84	(0.72, 0.97)	0.021
Past 30 day tobacco use	--	--	--	1.43	(1.27, 1.61)	0.000	1.32	(1.15, 1.50)	0.000
Current alcohol use	--	--	--	1.01	(0.91, 1.13)	0.818	1.01	(0.91, 1.13)	0.828
Current marijuana use	--	--	--	1.42	(1.24, 1.62)	0.000	1.42	(1.24, 1.63)	0.000
Current other drug use	--	--	--	2.26	(1.90, 2.68)	0.000	2.22	(1.87, 2.64)	0.000
Past 30 day menthol tobacco use	--	--	--	--	--	--	1.20	(1.02, 1.42)	0.029
Study Wave	1.02	(0.99, 1.04)	0.164	1.01	(0.99, 1.04)	0.208	1.01	(0.99, 1.03)	0.242

Note. *APR* Adjusted Poisson Regression. Wave is centered on first wave. Any individual missing data on a covariate was excluded

Those with missing data on alcohol, tobacco, marijuana, and other drug use (<2 %) were defined as non-current users

Table 3 shows correlates of current depression across all three models. Similar to the model for anxiety, adjusting for all covariates in the final model (Model 3), past 30-day use of any menthol tobacco product still emerged as a significant correlate of depression. Also, in the final model, Hispanic respondents and non-Black, non-Hispanic respondents were significantly more likely to report depression compared to White, non-Hispanic respondents in the final model. Similar to the model for anxiety, lower financial means was significantly associated with depression in the final model. Finally, after adjusting for all covariates in the final models, individuals with a high school education (vs. at least some college) were more likely to report depression, as were past 30-day tobacco users, current marijuana users, and current other drug users. Age and current alcohol use were not significant correlates of depression in the final model.

**Table 3:** Stepwise modified Poisson regression models of menthol use associated with current depression among n = 9720 (weighted) young adults

	Model 1			Model 2			Model 3		
	Demographics			Demographics, tobacco, and substance use			Demographics, tobacco, substance use, and menthol use		
Predictors	APR	(95 % CI)	*p*	APR	(95 % CI)	*p*	APR	(95 % CI)	*p*
Age									
18-24	Reference			Reference			Reference		
25-34	1.00	(0.88, 1.12)	0.948	1.01	(0.89, 1.13)	0.907	1.01	(0.89, 1.13)	0.898
Gender									
Male	Reference			Reference			Reference		
Female	1.18	(1.04, 1.33)	0.008	1.24	(1.10, 1.40)	0.000	1.23	(1.09, 1.39)	0.001
Race									
White, non-Hispanic	Reference			Reference			Reference		
Black, non-Hispanic	1.17	(0.97, 1.42)	0.097	1.18	(0.98, 1.41)	0.079	1.15	(0.96, 1.39)	0.136
Other, non-Hispanic	1.38	(1.12, 1.70)	0.002	1.39	(1.13, 1.71)	0.002	1.38	(1.12, 1.69)	0.002
Hispanic	1.18	(1.01, 1.37)	0.036	1.21	(1.05, 1.41)	0.009	1.21	(1.04, 1.40)	0.012
Education									
Less than high school	1.18	(0.98, 1.43)	0.083	1.10	(0.91, 1.31)	0.330	1.09	(0.91, 1.31)	0.366
High school	1.19	(1.04, 1.37)	0.011	1.15	(1.01, 1.32)	0.034	1.15	(1.01, 1.32)	0.033
At least some college	Reference			Reference			Reference		
Financial Situation									
Does not meet basic expenses	2.29	(1.92, 2.72)	0.000	2.11	(1.78, 2.51)	0.000	2.11	(1.78, 2.50)	0.000
Just meet basic expenses	1.48	(1.30, 1.68)	0.000	1.44	(1.27, 1.64)	0.000	1.44	(1.27, 1.64)	0.000
Meet needs with a little left	Reference			Reference			Reference		
Live comfortably	0.87	(0.73, 1.03)	0.097	0.87	(0.74, 1.02)	0.087	0.87	(0.74, 1.02)	0.086
Past 30 day tobacco use	--	--	--	1.33	(1.17, 1.51)	0.000	1.21	(1.04, 1.41)	0.015
Current alcohol use	--	--	--	0.96	(0.86, 1.08)	0.539	0.96	(0.86, 1.08)	0.521
Current marijuana use	--	--	--	1.48	(1.28, 1.71)	0.000	1.49	(1.29, 1.71)	0.000
Current other drug use	--	--	--	2.12	(1.75, 2.57)	0.000	2.07	(1.71, 2.52)	0.000
Past 30 day menthol tobacco use	--	--	--	--	--	--	1.24	(1.03, 1.50)	0.021
Study Wave	0.99	(0.96, 1.01)	0.213	0.98	(0.96, 1.01)	0.147	0.98	(0.96, 1.00)	0.121

Note. *APR* Adjusted Poisson Regression. Wave is centered on first wave. Any individual missing data on a covariate was excluded. Those with missing data on alcohol, tobacco, marijuana, and other drug use (<2 %) were defined as non-current users

## Discussion

Studies linking menthol tobacco use, beyond just cigarette smoking, to higher rates of depression/anxiety in young adults are scant. This study sought to uncover whether menthol tobacco-using young adults show certain mental health vulnerabilities relative to non-menthol users, after controlling for other covariates known to impact both mental health and tobacco use in this age group.

Bivariate analyses showed that current menthol-flavored tobacco users reported anxiety and depression at a higher prevalence compared to the full sample and to non-menthol tobacco users. Consistent with prior research, women, those with lower educational and financial means, and those currently using tobacco and other drugs were more likely to report anxiety and depression [64, 65]. Race was differentially associated with depression and anxiety, such that non-White respondents were most likely to report depression; however race was unrelated to anxiety. Alcohol use was not associated with either depression or anxiety, which we would not expect based on prior [34, 66]. Our findings may have been due to our assessment of alcohol use, which measured only general consumption in the past 30-days, but not consequences of use or heavy drinking; alcohol-related problems or indices of risky drinking behavior may be better predictors of mental health problems.

Stepwise modified Poisson regression models revealed that the odds of reporting anxiety and depression were nearly two and half times greater among those not meeting basic financial needs, relative to those meeting basic needs with some additional money left over. Further those currently using other drugs, such as cocaine, methamphetamine, or ecstasy, had the highest risk of anxiety and depression; nearly three times higher (for both outcomes) relative to respondents not using other drugs. Lastly, as we predicted, menthol tobacco use was associated with elevated risk for both mental health outcomes, after controlling for a number of other risk factors. This suggests there is something unique about menthol in tobacco products that links it to affective vulnerability. Such findings are concerning given the robust association between psychiatric distress, particularly depression, with more severe nicotine dependence, smoking persistence, and higher rates of smoking relapse among adult tobacco users [20, 67–70]. Thus, mental health may be one factor explaining why menthol smokers have worse cessation outcomes and greater nicotine dependence relative to non-menthol smokers in some studies [7]. This has important implications for incipient tobacco users with depression or anxiety. These individuals may start their tobacco use “careers” with menthol, but may find it exceedingly difficult to quit in later years because of the subjective rewarding effects of menthol [16], as well as the desire to self-medicate negative mood symptoms with nicotine [36, 40].

There could be several reasons why menthol is linked to mental health. First, the stimulating properties of nicotine, coupled with the positive sensory aspects of menthol, may relieve the negative moods and other aversive symptoms (i.e., weight gain, irritability, fatigue) that are common among individuals with depression or anxiety [36, 40]. If nicotine was the only mood-enhancing factor of tobacco use, then we would not have found differences between menthol and non-menthol tobacco users. These findings add to the literature, and suggest that menthol tobacco has distinct properties that drives its use among certain groups of users, and which may lead to more persistent use later on [12, 16, 17, 71]. Alternatively, individuals with an underlying vulnerability toward a variety of health-risk behaviors may self-select to use menthol tobacco. This may be because menthol flavoring is offered in a variety of different tobacco products [43] and vulnerable young adults are likely to try many substances of abuse, not just cigarettes [72]. A third reason could be that menthol is more likely to be sold in neighborhoods where socially and economically disadvantaged individuals reside, including those with mental health problems. For example, Young-Wolff and colleagues [27] found that tobacco retailer densities were twice as high in areas occupied by smokers with serious mental illness relative to the general population. Our findings showed that individuals with lower financial means and educational attainment were prone to mental health problems, and thus, the link between menthol use and mental health in young adults may be partially explained by tobacco marketing toward these lower income individuals. We did not assess the impact of tobacco marketing, nor neighborhood composition in this study.

There were several limitations of this study. First, we cannot conclude that menthol causes mental health problems due to the cross-sectional nature of the analyses. Similarly, we did not examine product switching, or the case in which someone could have been a menthol user at one time point, but no longer a menthol user at a subsequent time point; although prior studies indicate this is not common [1, 73]. Follow-up studies can examine whether age of menthol initiation predicts subsequent onset or progression of anxiety and depression. Second, this was a secondary analysis and we were limited to the measurement items within the dataset. Other factors not currently measured, such as neighborhood composition and social network factors, may impact rates of menthol use and mental health. Third, rates of anxiety and depression in the present study were higher than those reported in other national samples [21, 22]. This is likely due to the sensitivity of our 2-item measures. A more in-depth clinical assessment of depression, anxiety, and substance use features may be warranted [74]. Fourth, this study uses an existing online panel to recruit a large, national cohort of young adults. Although African American and Hispanic young adults were oversampled to ensure sufficient sample sizes, our sample may not be representative of the national population of young adults. Finally, we did not examine the impact of individual tobacco products (i.e.,e-cigarettes, LCCs, hookah) on mental health outcomes due to small sample sizes. Given the rising popularity of flavored tobacco use in young people [43], future work should examine product-specific links between menthol flavoring and mental health in this age group.

This study has several strengths. We are not aware of any national studies that have examined the link of menthol tobacco use, beyond menthol cigarette use, and mental health in young people. Of the two large-scale studies that have been published, both focused on adult populations and used either a general measure of psychiatric distress [28] or a crude measurement of well-being [30]. Further, both focused exclusively on menthol cigarette use, not tobacco use more generally. Examining a broad set of menthol tobacco product use, not just cigarette smoking, is particularly relevant to young people. Young adults have the highest rates of tobacco product use (cigarette and non-cigarette products) relative to any other age group and are highly likely to use a variety of different types of tobacco products [44]. Further, the use of flavored tobacco products, including menthol, is particularly prevalent in this age group [43].

## Conclusions

In summary, findings provide evidence that menthol is disproportionately used by vulnerable groups of young adult smokers, beyond the impact of other robust mental health and tobacco use risk factors. Menthol’s link to anxiety and depression, over and above non-mentholated tobacco product use, and the rising prevalence of menthol use in young adults makes the use of these mint-flavored tobacco products a significant public health concern. These findings have important implications for future policies and prevention campaigns aimed at deterring the uptake of menthol tobacco use among vulnerable and at-risk groups who have disproportionately high rates of use. Even though it is clear that menthol provides an “extra something” [75], and may contribute to mental health beyond just nicotine alone, the mechanisms underlying menthol’s link to depression and anxiety, nicotine addiction, and initiation and progression are not clear. Policies should be put in place to ban or further restrict the sale and marketing of menthol tobacco to youth and young people given these risks.

### Ethics approval

This study was approved by the Independent Investigational Review Board, Inc. for Waves 1-3 (Protocol #20036-007) and Chesapeake Institutional Review Board, Inc. for Waves 4-7 (Protocol #20036020). Online consent was collected from participants before survey self-administration.
